# Emerging threats and opportunities to managed bee species in European agricultural systems: a horizon scan

**DOI:** 10.1038/s41598-023-45279-w

**Published:** 2023-10-23

**Authors:** Bryony K. Willcox, Simon G. Potts, Mark J. F. Brown, Anne Alix, Yahya Al Naggar, Marie-Pierre Chauzat, Cecilia Costa, Antoine Gekière, Chris Hartfield, Fani Hatjina, Jessica L. Knapp, Vicente Martínez-López, Christian Maus, Teodor Metodiev, Francesco Nazzi, Julia Osterman, Risto Raimets, Verena Strobl, Annette Van Oystaeyen, Dimitry Wintermantel, Nikol Yovcheva, Deepa Senapathi

**Affiliations:** 1 Centre for Agri-Environmental Research, School of Agriculture, Policy and Development, University of Reading, Reading, RG6 6AR UK; 2 Department of Biological Sciences, Royal Holloway University of London, Egham, UK; 3 Corteva Agriscience, Regulatory and Stewardship Europe, Middle East and Africa, Abingdon, UK; 4 General Zoology, Institute for Biology, Martin Luther University Halle-Wittenberg, Hoher Weg 8, 06120 Halle (Saale), Germany; 5 Zoology Department, Faculty of Science, Tanta University, Tanta, 31527 Egypt; 6 Unit of Bee Research and Honey Production, Faculty of Science, King Khalid University, P.O. Box 9004, 61413 Abha, Saudi Arabia; 7 ANSES, Sophia Antipolis Laboratory, Unit of Honey Bee Pathology, 06902 Sophia Antipolis, France; 8 CREA Research Centre for Agriculture and Environment, 40128 Bologna, Italy; 9 Laboratory of Zoology, Research Institute for Biosciences, University of Mons, Mons, Belgium; 10 National Farmers’ Union, Agriculture House, Stoneleigh Park, Stoneleigh, Warwickshire CV8 2TZ UK; 11 Department of Apiculture, Institute of Animal Science, ELGO ‘DIMITRA’, 63200 Nea Moudania, Greece; 12 Department of Botany, School of Natural Sciences, Trinity College Dublin, Dublin 2, Ireland; 13 Department of Biology, Lund University, Lund, Sweden; 14 Department of Evolution, Ecology and Behaviour, Institute of Infection, Veterinary and Ecological Sciences, University of Liverpool, Liverpool, UK; 15 Department of Zoology and Physical Anthropology, Faculty of Veterinary, University of Murcia, 30100 Murcia, Spain; 16 Bayer AG, Alfred-Nobel-Str. 50, 40789 Monheim, Germany; 17 Pensoft Publishers, Sofia, Bulgaria; 18 Dipartimento di Scienze Agroalimentari, Ambientali e Animali, Università degli Studi di Udine, Udine, Italy; 19 National Biodiversity Future Center, Palermo, Italy; 20 Nature Conservation and Landscape Ecology, University of Freiburg, Tennenbacher Str. 4, 79106 Freiburg, Germany; 21 Gothenburg Global Biodiversity Centre, Gothenburg, Sweden; 22 Department of Biological and Environmental Sciences, University of Gothenburg, Gothenburg, Sweden; 23 Department of Plant Protection, Estonian University of Life Sciences, 51014 Tartu, Estonia; 24 Institute of Bee Health, Vetsuisse Faculty, University of Bern, Bern, Switzerland; 25 Biobest Group NV, Westerlo, Belgium

**Keywords:** Ecology, Environmental sciences

## Abstract

Managed bee species provide essential pollination services that contribute to food security worldwide. However, managed bees face a diverse array of threats and anticipating these, and potential opportunities to reduce risks, is essential for the sustainable management of pollination services. We conducted a horizon scanning exercise with 20 experts from across Europe to identify emerging threats and opportunities for managed bees in European agricultural systems. An initial 63 issues were identified, and this was shortlisted to 21 issues through the horizon scanning process. These ranged from local landscape-level management to geopolitical issues on a continental and global scale across seven broad themes—*Pesticides & pollutants, Technology, Management practices, Predators & parasites, Environmental stressors, Crop modification,* and *Political & trade influences*. While we conducted this horizon scan within a European context, the opportunities and threats identified will likely be relevant to other regions. A renewed research and policy focus, especially on the highest-ranking issues, is required to maximise the value of these opportunities and mitigate threats to maintain sustainable and healthy managed bee pollinators within agricultural systems.

## Introduction

Managed pollinators provide a wide range of benefits to society in terms of contributions to food security, farmer and beekeeper livelihoods, and social and cultural values^1^. Bees are important pollinators worldwide, with ~ 20,000 species; however, only 19 bee species are currently managed for crop pollination services^2^. In Europe, the main managed bee species are *Apis mellifera*, *Bombus terrestris*, and to a lesser extent, solitary bees such as those belonging to the genus *Osmia*^3^. Bees, along with other pollinators, face a range of threats including landscape modification, climate change, pests, pathogens, and agrochemicals^4–6^. While these issues are common across both wild and managed species, there may be other risks or opportunities that are specific to managed bees in a European agricultural context. Identifying these stressors or opportunities in a timely and effective manner can enable the development of effective policies and mitigation strategies across Europe (EU and national equivalents) to sustain healthy populations of managed bees.

Safeguarding European food security and promoting agricultural sustainability remains a prominent political ambition, driving the implementation of the European Green Deal and the Farm to Fork strategy^7,8^. Yet, current geopolitical instabilities and recovery from the worldwide COVID pandemic could potentially delay or even undermine many of the identified pathways to achieving these goals^9^. In hindsight, these issues might have been foreseeable, highlighting the importance of a forward scanning process to ensure policies are as preemptive as possible, rather than reactive. To make informed decisions, policymakers and practitioners need to anticipate the likely developments and their impact to understand and proactively develop preventative action plans. A systematic approach, such as routine horizon scanning, can provide the necessary insights to do this^10,11^, helping guide research priorities to generate actionable knowledge for policy and practice.

Managed pollinators are an important part of European food sustainability and are integral to the Farm to Fork strategy. To this end, we used a core expert group to horizon scan for potential threats and opportunities to managed bees in European agricultural systems over the next five to ten years.

## Results

**Figure 1:**
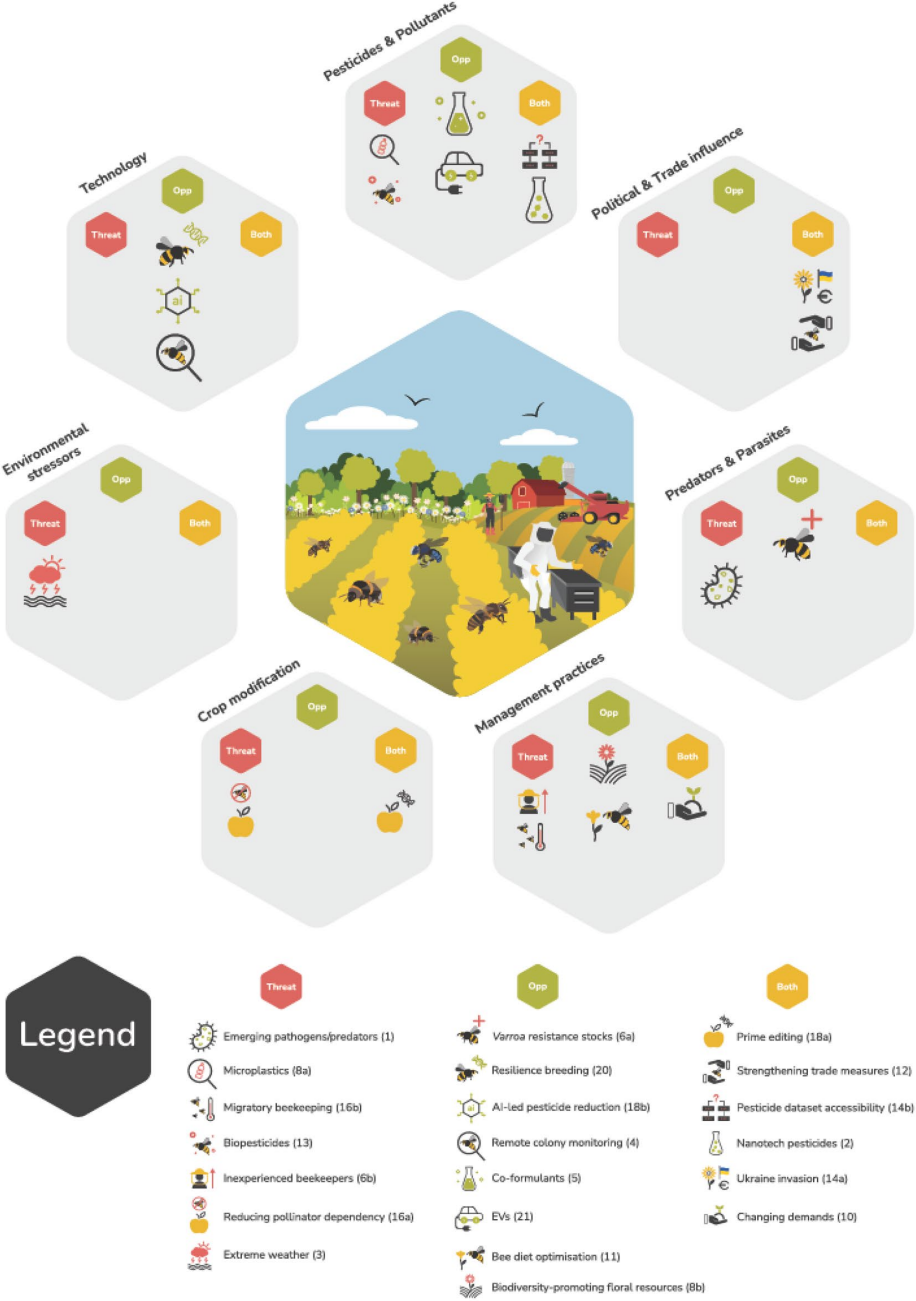
The 21 issues prioritized as a part of our 2022 horizon scan process and thematically grouped.

A summary for each of the 21 shortlisted issues follows (Fig. 1; Table 1). Issues are listed by whether they were identified as an opportunity, threat, or both. Issue rank order and broader theme are indicated in parentheses e.g., [*4*; *Technology*].

**Table 1:** The list of 21 issues prioritized as a part of our 2022 horizon scan process. Column ‘Issue Type’ refers to whether issues were determined to be a threat (T), opportunity (O) or both (B).

Rank order	Issue type	Topic	Median rank	
			1st round scoring	2nd round scoring
1	T	Increasing threat of emerging pathogens and predators	10	2
2	B	Nanotechnology-based pesticides (NBPs)	16	3.5
3	T	Extreme weather events	10.5	5
4	O	Greater availability of technology and automation to remotely monitor bee colony health	21	6.5
5	O	Co-formulants in agrochemical formulations and managed bee health	9.5	7
6a	O	Increase of varroa-resistant stocks of *Apis mellifera*	17.5	7.5
6b	T	Increase of inexperienced beekeepers	22.5	7.5
8a	T	Exposure to micro or nano plastics either alone or in combination with other stressors and transgenerational impacts on bees and bee health	20.5	8
8b	O	Agricultural policy to encourage biodiversity-promoting floral resources on arable land	20.5	8
10	B	Changing farm practice and timing of the demand for managed bees	21	9.5
11	O	Optimising diets of managed bees to develop better artificial diets and inform agri-environment schemes	16	10.5
12	B	Strengthening trade and biosecurity measures in the EU to better protect local managed bee populations, managed bee breeding and trade	16.5	11
13	T	Direct or indirect effects of biopesticides on bees	20.5	11.5
14a	B	Impact of Ukraine Invasion on the EU Common Agricultural Policy (rapid policy changes or delay of the green-deal due to Russian attack on Ukraine), food prices and agroecological transitions	20.5	12
14b	B	Accessibility of European pesticide exposure datasets	27	12
16a	T	Cutting pollinators out of food production	12.5	12.5
16b	T	Increase of migratory beekeeping	11.5	12.5
18a	B	Prime editing and genetically modified crops in Europe	23	13
18b	O	Artificial intelligence for disease, weed and pest control to reduce pesticide use in agroecosystems	23	13
20	O	Development of field instruments for evaluation of genetic markers to be used in breeding for resilience	21	17.5
21	O	Thermic vehicles and the hazardous pollutants they release will decrease in the coming years, does switching to electric vehicles represent an opportunity for managed bees?	22.5	18.5

### Opportunities

#### Greater availability of technology and automation to remotely monitor bee colony health [*4; Technology*]

The development of new techniques, to monitor and improve bee colony health status, based on artificial intelligence and deep learning has provided enormous recent advances in the field^12^. Advances include systems that track honey bees over hundreds of meters with high precision^13^, and new tools to monitor parameters such as duration and number of foraging trips (i.e., potential proxy for food flow) of individual solitary bees^14^. Furthermore, integration of disease and parasite prevalence with meteorological predictions and nectar flow information can provide the basis for important decision support tools for beekeepers, provided that the data is validated with appropriate field studies. A recent project attempted to integrate different types of data originating from diverse sources^15^, but further effort is required in this direction as currently data collection is highly unaggregated and diverse. Geographical information systems can also be used for supporting local and central authorities in decision-making processes relating to environmental planning^16^. The development of sensor technology, the spread of wireless infrastructures, and the increased ability to manage and model big data and provide predictions, could all represent an opportunity to interconnect apiaries across Europe and produce real-time predictions that could support decisions in the field.

#### Co-formulants in agrochemical formulations and managed bee health [*5; Pesticides & Pollutants*]

While co-formulants (i.e., ingredients added to active substances to produce the formulated product) are not expected to exert pesticidal impacts^17^, some were already shown to have lethal effects on honey bees in the early 1970s^18,19^ and additional concerns have been raised recently^20,21^. Current regulatory requirements list acute and chronic toxicity studies for formulations, which includes the testing of co-formulants in the context of the entire formulation^22^. A recent study confirmed that this requirement is justified by showing that different formulations of a herbicide varied in toxicity to bumble bees, due to differences in co-formulants rather than the active ingredient^23^. However, not all formulations are tested, and for those that are, testing can be quite limited^21^. Reinforcing the systematic study of formulant and formulation toxicity is therefore a potential opportunity to improve managed bee health. For example, if future research shows that specific co-formulants have potential impacts on managed bees, these could be removed or replaced by less impactful ingredients reducing a potential risk to managed bee health. Finally, a more in-depth knowledge of co-formulant toxicity could help to inform risk management and product labelling, and training for use that reduces exposure.

#### Increase of varroa-resistant stocks of *Apis mellifera* [*6a; Predators & Parasites*]

The significant negative impact of varroa mites on honey bees is well-established and widely recognised^24,25^. Most beekeeping operations strongly rely on chemical treatments to control mite populations; however, these can cause negative side effects and may become ineffective^26^. An alternative approach is to selectively enhance heritable honey bee traits for resistance or tolerance to the mite through breeding programs or select for naturally surviving untreated colonies. A recent review^27^ of studies on populations resistant or tolerant to varroa showed that in most cases, survival of both naturally and artificially selected populations is due to the expression of several traits (e.g., grooming, hygienic behaviour, varroa sensitive hygiene) that appear to collectively confer resilience to varroa infestation. Currently, around fifteen traits are recognised as regulatory traits that can be assessed in the field or in the lab^27^. However, a Europe-wide survey showed that despite huge demand, there is no well-established market for resistant stock in Europe, in part due to the increased cost of resistant stock and variable honey production benefits (i.e., resistant stock did not always produce more honey)^28^. The next ten years could represent a turning point, triggered by current concerns (e.g., increasing food security and declining wild pollinators), where breeding strategies and beekeeping management move towards the development of varroa resistant stocks.

#### Agricultural policy to encourage biodiversity-promoting floral resources on arable land [*8b; Management Practices*]

Ambitious sustainability goals within the European Green Deal^7^ and associated strategic policies such as the Biodiversity Strategy^29^, and the Nature Restoration Law^30^, have created a policy window for new biodiversity-promoting agricultural practices. "High-diversity landscape features" are a key component of the European Green deal and with the new CAP moving towards supporting biodiversity-friendly farming, opportunities have been created for biodiversity-promoting agricultural practices in Europe—called for by scientists^31,32^ and authorities^33^. Measures to achieve areas of ‘high diversity’ include implementing pollinator-friendly actions, such as the promotion of wild and cultivated flowers on large amounts of arable land^34,35^ and improving the quality of existing habitats to better meet the needs of managed bees and other pollinators^36^.

#### Optimising diets of managed bees to develop better artificial diets and inform agri-environment schemes [*11*;* Management Practices*]

The nutritional requirements of managed bees today may not be sufficiently met due to landscapes being increasingly characterized by agriculturally intensive cropping and monocultures^37^. The differences between what bees require and what their environment can provide, has contributed to the decline in managed bee populations in some countries (e.g., USA)^38^, and raises the questions of whether and how managed bees should be provided with supplemental food when nutritional deficits occur. Studies show that access to floral, and pollen, resource diversity provides amino acids and lipids that can support overall development, tolerance to parasites and immune system activity of bees^39–41^. This knowledge could be used to improve artificial diets and inform agri-environment schemes by selecting appropriate floral resource combinations to support pollinators and could accompany ongoing actions under the EU Biodiversity Strategy. For example, pollen of Asteraceae plants, including sunflowers, have been shown to reduce parasitic infection in managed bee species^42^. However, solely relying on Asteraceae pollen might not be sufficient, as it has a low protein content^43^, but if included in a pollen mix it could help improve pollinator health. Developing tailored seed mixtures to meet bee nutritional and health requirements could be a great opportunity in the next few years.

#### Artificial intelligence for disease, weed and pest control to reduce pesticide use in agroecosystems [*18b*;* Technology*]

Artificial Intelligence (AI) is the use of digital data and technology to fulfill specific operations such as weeding (using robots that can recognize weeds and remove them), or sensor equipped sprayers that allow direct application of a herbicide on to weeds only (reducing the volume of products sprayed by more than 50%^44^). It is estimated that one-third of global crop production is lost due to weed competition and another third due to pest and disease damage, with pesticides effective in combating these^45^. As early as the mid-1980s, AI for disease, weed and pest control was discussed^46^, and the first AI applications for crop production were developed^47^. The use of AI for disease and weed control is certainly expected to increase; however, even though AI solutions have already been used for over three decades in agriculture, their use to specifically reduce the risk to bees associated with pesticides is limited^48^. Nonetheless, it presents an opportunity to reduce potential risks to managed bee health.

#### Development of field instruments for evaluation of genetic markers to be used in breeding for resilience [*20*;* Technology*]

Biotechnology is advancing at a fast pace^49^, and recent advances could help to facilitate efforts to identify and select molecular markers that indicate the presence of certain resilience traits in honey bees. For instance, causative genes and proteins associated with resistance or tolerance could be developed as marker-assisted selection (MAS) tools for improving breeding stock at a large scale^50,51^. In addition, DNA-based technologies have become more affordable over the last decades, so the financial aspects may not necessarily be prohibitive. Relatively cheap single nucleotide polymorphism (SNP)-based assays have already been developed for some traits linked to resilience^52^. Portable PCR tools are already in use^53^, and it is feasible to foresee portable genetic marker kits that could directly be used in the field and assist beekeepers in selecting colonies with traits linked to resilience (to parasites, to drought, to higher temperatures). However, this potential is offset by various issues including the differing suites of genes underlying resilience and sensitivity to stressors identified in different honey bee populations^25^.

#### Thermic vehicles and the hazardous pollutants they release will decrease in the coming years [*21*;* Pesticides* & *Pollutants*]

The opportunity arising from a shift from thermic to electric vehicles may be considered a relatively new issue. The global trend in electric vehicles suggests we will move from around a 5–10% market share in 2022 to a 25–50% share (depending upon region) by 2030^54^. The expectation is that the pressures on managed pollinators from pollutants from vehicles, in general, will be reduced, although it does not prevent all risks (e.g., turbulence and metals in dust) associated with road pollution^55^. Given the amount of land taken up by areas such as road verges (~ 270,000 km^2^)^56^, a proportion of which would be visited by bees, this is not an insignificant change. The situation is complex (e.g., environmental footprint of rare metal extraction) and hard to quantify, though qualitatively, the switch to electric vehicles would likely be an improvement.

### Threats

#### Increasing threat of emerging predators and pathogens [*1*; *Predators & Pathogens*]

The spread of non-native and invasive species and the emergence of novel pathogens, variants of existing ones and shifting modes of transmission are a continuing threat to managed bee populations^57–59^. For example, a recent modelling study showed that the steady increase in alien species belonging to different taxa observed in the last fifty years will not slow down in the near future in all continents including Europe^60^. Europe may become a suitable niche for new (e.g., *Vespa mandarinia*^61^) and spreading (e.g., *Vespa orientalis*^62–64^ and *Aethina tumida*^65^) species, thus adding to the pressure from current invasives (e.g., *Vespa velutina*^66^). Furthermore, pathogen transfers between honey bees and invasive species have been found, underlining that impacts on honey bee populations may be direct (i.e., predation) and indirect (i.e., pathogen dynamic)^67^. Additionally, any potential shift in virus transmission mode (e.g., from faecal/food-oral to vector mediated) could pose a future threat to bees and apiculture^57,68^. Therefore, it is likely that both the number of invasive predators and the impact from pathogens will continue to grow in the next ten years increasing the burden posed to managed bees.

#### Extreme weather events [*3*; *Environmental Stressors*]

The impact of some extreme weather and climatic events on pollinator communities is well-characterized in the literature^69–71^. However, the significance of these events, including those that are less well-characterized (e.g., extreme frost events), and how such events might interact with other drivers of decline to exacerbate negative impacts on managed bee populations across Europe, is less well understood. The impact of extreme temperature and heatwaves are already emerging^72,73^, and there is further anecdotal evidence that the summer heatwaves of 2022 in France affected egg-laying in honey bees during *Robinia pseudoacacia* nectar flow and severe spring rainfall in Spain led to colony collapse due to lack of foraging resources (anecdotal communications gathered by horizon scan experts). Interactions between extreme climatic events and other drivers of decline are a significant threat in the foreseeable future.

#### Increasing numbers of inexperienced beekeepers [*6b*; *Management Practices*]

Beekeeper experience is a key factor in determining responses to honey bee health issues^74^, and an increase in the number of inexperienced beekeepers has been identified as an emerging threat to bee health. Several studies at a pan-European level have found that beekeeper background and apicultural practices are major drivers of honey bee colony losses^75,76^. Inexperienced beekeepers with small apiaries experience up to double the winter mortality rate compared to experienced beekeepers, possibly due to inadequate disease control^77^. Sick colonies can also favour the spread of pathogens within *Apis mellifera* due to typical honey bee behaviour (robbing, swarming) and possibly also across other bee species^78^.

#### Exposure to micro- or nano-plastics either alone or in combination with other stressors and transgenerational impacts on bees and bee health [*8a*; *Pesticides & Pollutants*]

Micro-plastics (MPs) (plastics < 5 mm, including nano-plastics which are < 0.1 μm) have been identified as an emerging threat in terrestrial systems^79^. MPs are readily absorbed into plants from the soil^80^, and bee bodies through contaminated food under laboratory conditions^81^; they can also absorb pollutants such as pesticides acting as a source and sink of environmental contaminants^82^. MPs can increase honey bee mortality (albeit only at high concentrations^83^), decrease feeding rate and body weight^84^, change the diversity of gut biota and gene expression related to oxidative damage, detoxification, and immunity, and increase worker susceptibility to antibiotics^82^. MPs likely interact with other environmental stressors, and co-occurrences are highly likely in agricultural landscapes; for example, honey bees showed higher mortality to viral infection when exposed to MPs^85^. More research to monitor MPs (e.g., http://www.insignia-bee.eu) is needed to generalise exposure patterns, i.e., across food webs (nectar and pollen), between bee species and in different landscape contexts, to provide essential information for their monitoring and management^82,86^. Given MPs are already ubiquitous in the environment^87^ and are poorly understood in the context of managed bees^88^ there is the potential for them to be a significant threat to managed bees.

#### Direct and indirect effects of biopesticides on bees [*13*; *Pesticides* & *Pollutants*]

Biopesticides include a broad range of products, including natural (or nature identical) chemical substances, plant or animal extracts, pheromones or semiochemicals, untransformed inorganic pesticides and microorganisms (e.g., bacteria, viruses, or fungi). A recent update in the EU Regulations has clarified the data requirements and approval criteria for a subcategory of biopesticides (microorganisms)^89^, yet concerns remain around the risk assessment of biopesticides in general. In the case of semiochemicals, inorganics and nature-identical chemicals that are usually the sole active component in a formulation, risk assessments are well established. However, for complex mixtures or microorganisms that typically exert activity as an organism plus secondary active metabolites, testing methods are still evolving and, in some instances, may not be developed enough to provide clear results^90,91^. Without new standardized testing methods to address potential non-intentional effects of biopesticide active substances and their formulations on managed bees, biopesticides could represent a significant threat.

#### Increase of migratory beekeeping [*16b*;* Management Practices*]

More frequent droughts and severe heat waves will likely contribute to an increase in migratory beekeeping, with increases expected in terms of the proportion of hives relocated and the distance travelled. Additionally, European policies provide subsidies for migratory beekeeping, as a means of providing ecosystem services to marginal areas^92^. Recent studies, however, suggest that migratory beekeeping leads to increased disease risk^93^ (although see Bartlett et al.^94^), genetic introgression^95,96^ and may affect local pollinator biodiversity^97^. Given the importance of locally adapted genotypes in Europe^98^ and the threats posed by disease, increases in migratory beekeeping could have a high negative impact on European honey bee health.

#### Cutting pollinators out of food production [*16a*; *Crop Modification*]

Excluding pollinators from food production continues to be a threat to the sustainability of managed bee populations, through plant breeding and cultivation practices. For example, methods to promote parthenocarpy (fruit set in the absence of fertilisation), such as genetic modification, hormone application and selective breeding, may reduce the need for pollinators in many horticultural crops^99^. Whilst reducing our dependence on pollinators may allow growers to extend their growing seasons, it could remove our imperative to utilise them^10^. This may have unintended consequences for commercial beekeepers and apiaries, to ultimately affect the pollination of non-parthenocarpic pollinator-dependent crops such as seed and nut crops and wild plants.

### Both a threat and an opportunity

#### Nanotechnology-based pesticides (NBPs) [*2*; *Pesticides* & *Pollutants*]

Nanotechnology can modify a pesticide's solubility, stability, and efficacy to improve crop protection^100^. However, this process changes NBPs' environmental fate and behaviour, and this emerging technology has outpaced our understanding of how NBPs may affect pollinators^101,102^. NBPs may be an opportunity for managed bees as their stability and controlled-release mechanisms increase efficiency to reduce the chemical required on crops^103^. Only one study has explored the effect of NBPs on pollinators, showing that a pyrethrum extract in a nanocarrier was safer than a traditional pyrethrum extract^104^. However, like traditional pesticides, NBPs may threaten managed bees and other non-target organisms through toxicity, yet virtually no data exist to test this^105^. Indeed, the structure of NBPs, which is similar to pollen, means that bees are adapted to collect and move NBPs, resulting in exposure, and no studies have explored bees' exposure to NBPs^101^. NBPs are rapidly evolving, poorly understood, and likely to substantially impact managed bees in agricultural landscapes.

#### Changing farm practice and timing of the demand for managed bees [*10*; *Management Practices*]

Among the EU Green Deal strategic policies, the development of Sustainable Food Systems foresees a significant change in food production schemes and practices^8^, which may either pose an opportunity or a threat depending on the context and the practices recommended or adopted. Opportunities may exist through fulfilling global strategic moves to diverse crop production, less dependence on global markets and increased connection to local production sources, and more sustainable approaches taken with respect to the use of water and energy resources or the use of land^16^. For example, recent research has highlighted the potential benefits of crop diversification for pollinators while keeping crop yield stable^106^, although crop diversity also drives the frequency and intensity of pesticide use^107^. Refining effective agricultural best-practices, such as selecting optimal seed-mixes for floral strips, may also increase the benefits for pollinators and offer further opportunities^108^. These practices would operate alongside changes triggered by adaptations to climate change, which the policies are trying to tackle. In this context, changes that may negatively impact managed bees will be observed in crop availability, growing and flowering seasons, with concomitant impacts on the need for managed pollinators in space and time to meet crop pollination demands, and honey production.

#### Strengthening trade and biosecurity measures in Europe to better protect local managed bee populations, managed bee breeding and trade [*12*;* Political *& *Trade Influences*]

The lack of limitations on the trade and movement of managed bees has benefitted disease spread and has been causing genetic erosion of local bee populations^93,109,110^, ultimately resulting in the loss of traits involved in bee resilience. Currently, bees fall under several regulations at European level for importations^111–113^, and only honey bee queens and bumble bees are permitted to enter the EU, subject to health requirements. Health requirements include checking for signs of small hive beetle (*Aethina tumida*), mite (*Tropilaelaps* spp. and *Varroa* spp.) and bacterial (*Paenibacillus* larvae) infestations, however there are no regulations regarding other pathogens or trade magnitude^114^. To prevent genetic erosion of local bee populations, subspecies of bees need to be included in regulations. This is particularly pertinent given genotype-environment interactions are described as underlying the complex relationships between local populations of honey bees, landscape, infection, and parasites (particularly *Varroa* spp., viruses and *Nosema* spp.). Furthermore, regulations for solitary bee trade should also be introduced. Without these changes the threat to managed bee populations will continue, however, there is an opportunity for EU legislators to include genetic diversity protection of managed bees in the CAP strategy and more specifically in the National Apiculture Programmes. In this way, trade and biosecurity measures can contribute to the protection of local managed bee populations from genetic introgression, as well as from the spread of diseases.

#### Impact of war in Ukraine on the EU Common Agricultural Policy, food prices and agroecological transitions [*14a*; *Political *& *Trade Influences*]

The Russian invasion of Ukraine has significantly affected the import and export of crops and grains that impact food security. In response, the European Commission^115^ has presented a range of short-term and medium-term actions to enhance global food security and to support farmers. Impacts of the war in Ukraine on the agricultural policy of Europe may be both a threat and an opportunity for managed bees. For example, the recent decision to allow the tillage of fallow lands to palliate food shortages due to the conflict may lead to a reduction in the uptake of agri-environment type measures (e.g., wildflower strips) that benefit bees. However, if alternative crops which are mass flowering, such as clover or sunflower, are planted then at least for the flowering period there could be a benefit for bees^116^.

#### Accessibility of European pesticide exposure datasets [*14b*; *Pesticides* & *Pollutants*]

Researchers, particularly ecotoxicologists, need precise information on pesticide use in the landscape. While the EU Pesticides Database^117^ provides information such as active substances used in plant protection products or Maximum Residue Levels (MRLs) in food products, it does not provide information on spatial and temporal patterns of use of commercial products across Europe. There are two main sources of information for pesticide use at European level: the Common Agriculture Policy (CAP) dataset and data produced to comply with the regulations on statistics on pesticides^118^. Currently, these datasets are not readily accessible to the public. Although attempts to address these issues in the regulatory framework are underway (e.g., through the requirement for records of pesticide use to be kept by farmers^118^), data from the different European countries are not aggregated in a single database and efforts still need to be made to standardise data collection and collation across Member States.

#### Prime editing and genetically modified crops in Europe [*18a*; *Crop Modification*]

The EU currently has extensive limits on the use and development of GM crops. Member States are seeking new regulatory frameworks to make EU research institutions competitive at an international level^119^. Along with base editing, prime editing is a relatively new genomic technique based on the CRISPR–Cas9 system^120^. This presents an opportunity, as the first prime edited plant species could be commercially available in 2023^121^ joining a number of genetically modified (GM) crops already utilised worldwide^122^. While pest-resistant crops benefit non-target organisms due to reductions in insecticide use^123,124^, herbicide-resistant crops favour the use of herbicides around valuable crops. This extensive use of herbicides eliminates non-cultivated plants around crop fields that are known to be beneficial to pollinators^125,126^. Impacts of other GM crop types, such as abiotic stress-resistant, disease-tolerant, and nutritionally improved crops, have not yet been assessed on managed bees but could pose both a threat and an opportunity.

## Concluding remarks

Through the horizon scanning process 21 issues with the potential to impact managed bees in European agricultural systems were prioritised, from an initial 63. These fell under seven broader themes (Fig. 1): *Pesticides & pollutants, Technology, Management practices, Predators & parasites, Environmental stressors, Crop modification* and *Political & trade influences.*

A consistent point raised across multiple issues under the theme of *Pesticides & pollutants* was a current dearth of knowledge on the impact on managed bee populations. Examples include the threat posed by microplastic accumulation and its movement through the food chain, whether the fast-paced emergence of nanotechnology-based pesticides will provide threats or opportunities, or the benefits in transitioning from thermic to electric vehicles. For microplastics, current EU-funded research projects (e.g., www.insignia-bee.eu) are beginning to quantify their impact on various aspects of managed bee health, and with EU policies in place set to ban all single use plastics^127^, these results will be best placed to inform future monitoring activities. There was also a recognition of the need to support EU pesticide use and risk reduction policies, through recommendations on how to reduce risks from co-formulants and microorganisms used as biopesticides and providing standardised data on the spatial and temporal use of commercial pesticide products across the Member States.

Three opportunities prioritised in this scan fell under the theme of *Technology*. These ranged from remotely monitoring bee health and evaluating genetic markers in the field to the use of artificial intelligence in reducing pesticide use in agriculture. Rapid advancements in biotechnology and available tools are facilitating in-field monitoring and evaluation capabilities, however rapid adoption is key for these tools to be effective in beekeeper practices in real life.

Two issues were prioritised under the theme of *Crop modification*. The key aspect for both of these issues, which included cutting pollinators out of food production through a shift towards parthenocarpic crops and the uncertainty surrounding newer genomic techniques such as prime editing, is the lack of assessment on the impact on managed bees.

The threat to managed bees from extreme weather events was the only issue to fall under the theme of *Environmental stressors*. The impacts of well-characterised events, such as heat waves and drought, are already impacting bees and beekeeping practices. However, the potential threat to managed bees from interactions between extreme weather events (including less well characterised events such as frosts) and other stressors (e.g., pesticides and parasites) was recognised as a high priority area for research and should be considered in future policy outlooks.

Several issues resulting from changes to various *Management practices* were raised through this horizon scan process. Two key opportunities to support managed bee diets were highlighted, these included research-driven bee diet optimisation with the potential to lead to the creation of tailored seed mixes to meet nutritional requirements. These could then be utilised for implementing diverse on-farm floral resources, which has gained further policy support under the sustainability goals of the European Green Deal. In contrast, increases in both inexperienced beekeepers and migratory beekeeping practices were recognised as emerging threats with the potential to impact on managed bee health through higher disease prevalence and genetic introgression. Lastly, uncertainty around the impact of changing farm practices on managed bees was recognised, with both opportunities and threats foreseeable dependent upon the context of the situation and the practices adopted.

The continually changing threat from invasive predators and emerging pathogens across Europe was the most highly ranked issue in this horizon scan and was one of two issues to come under the theme of *Predators & parasites*. The second was the opportunity around the development of *Varroa* resistant stocks, with the next few years recognised as a potential turning point for this issue.

Finally, two issues were raised that fell under the theme of *Political and trade influence*. The European Commission response to recent geopolitical developments, such as the war on Ukraine, was raised here, particularly the uncertainty around the impact on managed bees of short- and medium-term actions aimed at supporting farmers and food security that may negate bee beneficial practices. Alongside the uncertainty of rapid policy changes in response to ongoing geopolitical issues was a recognition of the need to strengthen trade regulations to better protect managed bee populations.

Given the accelerating pace of technology, trajectory for current policy development and geopolitical crises we highlight the need to repeat this exercise in 5 years’ time.

## Methods

We followed a horizon scanning approach based on a modified Delphi technique and previous horizon scans^10,11^. A core group of 20 experts from nine European countries undertook the scanning exercise. Participants were members of a wider consortium collaborating on the EU-funded project, PoshBee—Pan-European Assessment, Monitoring and Mitigation of Stressors on the Health of Bees (http://www.poshbee.eu). Experts were affiliated with research institutes, universities, government and non-government organisations and industry. In this scan, we consider both policy and practice contexts, and issues in the EU, the UK, Switzerland, and Norway.

Each expert was encouraged to consult with their networks to collect up to 5 potential horizon issues. The aim was to identify poorly known issues that could have a substantial positive or negative impact on managed bees (e.g., *Apis mellifera*, *Bombus* spp., *Osmia* spp.) in European agricultural systems over the next 10 years.

Initial submissions that dealt with similar issues were grouped together by topic area and direction of impact (threat or opportunity), to be scored collectively. A list of 63 issues, including references, was compiled, and sent out to the core expert group to complete a first round of anonymous scoring (Table 1). Issues were scored from 1 (well known, unlikely to have a substantial impact on pollinators) to 100 (poorly known, likely to have a substantial impact on pollinators) following the methods adopted by Brown et al.^10^. From this first round of scoring, we produced a ranked list of issues for each participant and then calculated the median rank for each horizon issue (Table 1). The 20 top ranking issues, along with comments and references, were kept as a reasonable number which could be assessed in depth in the next stages of the process. After this initial scoring participants were given the opportunity to retain any issues they felt strongly should have been included. One issue was retained by this process, therefore there were 21 issues in total (Fig. 1; highlighted in Table 1).

Based on their established domain knowledge two experts were assigned to each of the 21 issues to play the role of cynic and to further investigate their novelty, likelihood of emergence, and whether the impact on managed pollinators would be a threat, opportunity, or potentially both. Experts were not assigned to issues they had originally proposed. Experts wrote a short report on their assigned issues that included a summary of the current knowledge and evidence for why it was likely, or not, to be a significant threat or opportunity over the next decade. These reports were then compiled and shared with the group (authorship of individual reports was not revealed to the group) prior to the workshop discussion. To reduce biases due to reader fatigue the order of these short reports in the compiled document was reversed for half the participants.

An online workshop, with 16 experts in attendance, was held in July 2022. Each of the 21 issues was discussed, and following each discussion, experts privately re-scored the issue between 1 and 100, as previously described. The four experts unable to attend the workshop were sent detailed accounts of the discussions that took place and were asked to re-score each issue after reading these accounts.

### Supplementary Information

Supplementary Table 1.

## Data Availability

All data generated or analysed during this study are included in this published article (and its supplementary information files).

## References

[1] Potts, S. G. *et al. The Assessment Report on Pollinators, Pollination and Food Production: Summary for Policymakers* (Secretariat of the Intergovernmental Science-Policy Platform on Biodiversity and Ecosystem Services, 2016).

[2] Osterman J, Aizen MA, Biesmeijer JC, Bosch J, Howlett BG, Inouye DW, Jung C, Martins DJ, Medel R, Pauw A, Seymour CL (2021). Global trends in the number and diversity of managed pollinator species. Agric. Ecosyst. Environ..

[3] Osterman J, Landaverde-González P, Garratt MP, Gee M, Mandelik Y, Langowska A, Minarro M, Cole LJ, Eeraerts M, Bevk D, Avrech O (2021). On-farm experiences shape farmer knowledge, perceptions of pollinators, and management practices. Global Ecol. Conserv..

[4] Dicks LV, Breeze TD, Ngo HT, Senapathi D, An J, Aizen MA, Basu P, Buchori D, Galetto L, Garibaldi LA, Gemmill-Herren B (2021). A global-scale expert assessment of drivers and risks associated with pollinator decline. Nat. Ecol. Evol..

[5] Decourtye A, Alaux C, Le Conte Y, Henry M (2019). Toward the protection of bees and pollination under global change: Present and future perspectives in a challenging applied science. Curr. Opin. Insect Sci..

[6] Knapp, J. L., Nicholson, C. C., Jonsson, O., de Miranda, J. R. & Rundlöf, M. Ecological traits interact with landscape context to determine bees’ pesticide risk. *Nat. Ecol. Evol.* 1–10 (2023). 10.1038/s41559-023-01990-5 PMC10089916 36849537

[7] European Commission. Communication from the Commission to the European Parliament, The European Council, The Council, The European Economic and Social Committee and the Committee of the Regions The European Green Deal. Brussels, 11.12.2019, COM (2019) 640 final.

[8] European Commission. Communication from the Commission to the European Parliament, the Council, The European Economic and Social Committee and the Committee of the regions a Farm to Fork Strategy for a fair, healthy and environmentally-friendly food system. Brussels, 20.5.2020, COM (2020) 381 final.

[9] Morales MB, Díaz M, Giralt D, Sardà-Palomera F, Traba J, Mougeot F, Serrano D, Mañosa S, Gaba S, Moreira F, Pärt T (2022). Protect European green agricultural policies for future food security. Commun. Earth Environ..

[10] Brown MJ, Dicks LV, Paxton RJ, Baldock KC, Barron AB, Chauzat MP, Freitas BM, Goulson D, Jepsen S, Kremen C, Li J (2016). A horizon scan of future threats and opportunities for pollinators and pollination. PeerJ.

[11] Sutherland WJ, Butchart SH, Connor B, Culshaw C, Dicks LV, Dinsdale J, Doran H, Entwistle AC, Fleishman E, Gibbons DW, Jiang Z (2018). A 2018 horizon scan of emerging issues for global conservation and biological diversity. Trends Ecol. Evol..

[12] Odemer R (2022). Approaches, challenges and recent advances in automated bee counting devices: A review. Ann. Appl. Biol..

[13] Vo-Doan, T. T., & Straw, A. D. Millisecond insect tracking system. (2020). arXiv preprint arXiv:2002.12100.

[14] Knauer AC, Gallmann J, Albrecht M (2022). Bee Tracker—An open-source machine learning-based video analysis software for the assessment of nesting and foraging performance of cavity-nesting solitary bees. Ecol. Evol..

[15] Simón Delso N, Sušanj G, Salazar Abello A (2021). The EU Bee Partnership (EUBP) Prototype Platform: Data model description. EFSA Support. Publ..

[16] Marnasidis S, Kantartzis A, Malesios C, Hatjina F, Arabatzis G, Verikouki E (2021). Mapping priority areas for apiculture development with the use of geographical information systems. Agriculture.

[17] Mesnage R, Antoniou MN (2018). Ignoring adjuvant toxicity falsifies the safety profile of commercial pesticides. Front. Public Health.

[18] Moffett JO, Morton HL, MacDonald RH (1972). Toxicity of some herbicidal sprays to honey bees. J. Econ. Entomol..

[19] Moffett JO, Morton HL (1975). Repellency of surfactants to honey bees. Environ. Entomol..

[20] Mullin CA, Chen J, Fine JD, Frazier MT, Frazier JL (2015). The formulation makes the honey bee poison. Pesticide Biochem. Physiol..

[21] Straw EA, Thompson LJ, Leadbeater E, Brown MJF (2022). ‘Inert’ ingredients are understudied, potentially dangerous to bees and deserve more research attention. Proc. R. Soc. B.

[22] European Commission. Commission Regulation (EU) No 283/2013 of 1 March 2013 setting out the data requirements for active substances, in accordance with Regulation (EC) No 1107/2009 of the European Parliament and of the Council concerning the placing of plant protection products on the market (2013).

[23] Straw EA, Carpentier EN, Brown MJF (2021). Roundup causes high levels of mortality following contact exposure in bumble bees. J. Appl. Ecol..

[24] Le Conte Y, Ellis M, Ritter W (2010). Varroa mites and honey bee health: Can Varroa explain part of the colony losses?. Apidologie.

[25] Nazzi F, Brown SP, Annoscia D, Del Piccolo F, Di Prisco G, Varricchio P, Della Vedova G, Cattonaro F, Caprio E, Pennacchio F (2012). Synergistic parasite-pathogen interactions mediated by host immunity can drive the collapse of honeybee colonies. PLoS Pathog..

[26] Milani N (1999). The resistance of *Varroa **jacobsoni* Oud. to acaricides. Apidologie.

[27] Mondet F, Beaurepaire A, McAfee A, Locke B, Alaux C, Blanchard S, Danka B, Le Conte Y (2020). Honey bee survival mechanisms against the parasite *Varroa destructor*: A systematic review of phenotypic and genomic research efforts. Int. J. Parasitol..

[28] Buechler R, Uzunov A, Costa C, Meixner M, Le Conte Y, Mondet F, Kovacic M, Andonov S, Carreck NL, Dimitrov L, Basso B, Bienkowska M, Dall’Olio R, Hatjina F, Wirtz U (2022). EurBeST—A pilot study testing varroa resistant bees under commercial beekeeping conditions. Am. Bee J..

[29] European Commission. Communication from the Commission to the European Parliament, the Council, the European Economic and Social Committee and the Committee of the Regions, EU biodiversity strategy for 2030: Bringing nature back into our lives. Brussels, 20.5.2020, COM (2020) 380 final.

[30] European Commission. Proposal for a REGULATION OF THE EUROPEAN PARLIAMENT AND OF THE COUNCIL on nature restoration. Brussels, 22.6.2022, COM (2022) 304 final.

[31] IPBES. The assessment report of the Intergovernmental Science-Policy Platform on Biodiversity and Ecosystem Services on pollinators, pollination and food production. In (eds Potts, S. G. *et al.*). (Secretariat of the Intergovernmental Science-Policy Platform on Biodiversity and Ecosystem Services, 2016).

[32] Lundin O, Rundlöf M, Jonsson M, Bommarco R, Williams NM (2021). Integrated pest and pollinator management–expanding the concept. Front. Ecol. Environ..

[33] EEA. The European environment—state and outlook 2020: Knowledge for transition to a sustainable Europe. European Environment Agency (2020) https://www.eea.europa.eu/soer (Accessed 20 Mar 2023).

[34] Scheper J, Holzschuh A, Kuussaari M, Potts SG, Rundlöf M, Smith HG, Kleijn D (2013). Environmental factors driving the effectiveness of European agri-environmental measures in mitigating pollinator loss—A meta-analysis. Ecol. Lett..

[35] Jachuła J, Denisow B, Wrzesień M, Ziółkowska E (2022). The need for weeds: Man-made, non-cropped habitats complement crops and natural habitats in providing honey bees and bumble bees with pollen resources. Sci. Total Environ..

[36] Baden-Böhm F, Thiele J, Dauber J (2022). Response of honeybee colony size to flower strips in agricultural landscapes depends on areal proportion, spatial distribution and plant composition. Basic Appl. Ecol..

[37] Naug D (2009). Nutritional stress due to habitat loss may explain recent honeybee colony collapses. Biol. Conserv..

[38] Smart MD, Pettis JS, Euliss N, Spivak MS (2016). Land use in the Northern Great Plains region of the US influences the survival and productivity of honey bee colonies. Agric. Ecosyst. Environ..

[39] Alaux C, Ducloz F, Crauser D, Le Conte Y (2010). Diet effects on honeybee immunocompetence. Biol. Lett..

[40] Huang Z (2012). Pollen nutrition affects honey bee stress resistance. Terr. Arthropod Rev..

[41] Annoscia D, Zanni V, Galbraith D, Quirici A, Grozinger C, Bortolomeazzi R, Nazzi F (2017). Elucidating the mechanisms underlying the beneficial health effects of dietary pollen on honey bees (*Apis mellifera*) infested by Varroa mite ectoparasites. Sci. Rep..

[42] Palmer-Young EC, Malfi R, Zhou Y, Joyce B, Whitehead H, Van Wyk JI, Baylis K, Grubbs K, Boncristiani DL, Evans JD, Irwin RE (2023). Sunflower-associated reductions in Varroa mite infestation of honey bee colonies. J. Econ. Entomol..

[43] Pamminger T, Becker R, Himmelreich S, Schneider CW, Bergtold M (2019). Pollen report: Quantitative review of pollen crude protein concentrations offered by bee pollinated flowers in agricultural and non-agricultural landscapes. PeerJ.

[44] Dörr J, Nachtmann M (2022). Digital transformation for sustainable agriculture. Handbook Digital Farming.

[45] Zhang P, Guo Z, Ullah S, Melagraki G, Afantitis A, Lynch I (2021). Nanotechnology and artificial intelligence to enable sustainable and precision agriculture. Nat. Plants.

[46] McKinion JM, Lemmon HE (1985). Expert systems for agriculture. Comput. Electron. Agric..

[47] Jha K, Doshi A, Patel P, Shah M (2019). A comprehensive review on automation in agriculture using artificial intelligence. Artif. Intell. Agric..

[48] Zhang W (2018). Global pesticide use: Profile, trend, cost/benefit and more. Proc. Int. Acad. Ecol. Environ. Sci..

[49] Cornelissen M, Małyska A, Nanda AK, Lankhorst RK, Parry MA, Saltenis VR, Pribil M, Nacry P, Inzé D, Baekelandt A (2021). Biotechnology for tomorrow’s world: Scenarios to guide directions for future innovation. Trends Biotechnol..

[50] Grozinger CM, Robinson GE (2015). The power and promise of applying genomics to honey bee health. Curr. Opin. Insect Sci..

[51] Guarna MM, Hoover SE, Huxter E, Higo H, Moon KM, Domanski D, Bixby ME, Melathopoulos AP, Ibrahim A, Peirson M, Desai S (2017). Peptide biomarkers used for the selective breeding of a complex polygenic trait in honey bees. Sci. Rep..

[52] Jones JC, Du ZG, Bernstein R, Meyer M, Hoppe A, Schilling E, Ableitner M, Juling K, Dick R, Strauss AS, Bienefeld K (2020). Tool for genomic selection and breeding to evolutionary adaptation: Development of a 100K single nucleotide polymorphism array for the honey bee. Ecol. Evol..

[53] Marx V (2015). PCR heads into the field. Nat. Methods.

[54] Deloitte (2020). https://www2.deloitte.com/uk/en/insights/focus/future-of-mobility/electric-vehicle-trends-2030.html.

[55] Phillips BB, Bullock JM, Gaston KJ, Hudson-Edwards KA, Bamford M, Cruse D, Dicks LV, Falagan C, Wallace C, Osborne JL (2021). Impacts of multiple pollutants on pollinator activity in road verges. J. Appl. Ecol..

[56] Phillips BB, Bullock JM, Osborne JL, Gaston KJ (2020). Ecosystem service provision by road verges. J. Appl. Ecol..

[57] Remnant EJ, Mather N, Gillard TL, Yagound B, Beekman M (2019). Direct transmission by injection affects competition among RNA viruses in honeybees. Proc. R. Soc. B.

[58] Requier F, Rome Q, Chiron G, Decante D, Marion S, Menard M, Muller F, Villemant C, Henry M (2019). Predation of the invasive Asian hornet affects foraging activity and survival probability of honey bees in Western Europe. J. Pest Sci..

[59] Proesmans W, Albrecht M, Gajda A, Neumann P, Paxton RJ, Pioz M, Polzin C, Schweiger O, Settele J, Szentgyörgyi H, Thulke HH (2021). Pathways for novel epidemiology: Plant–pollinator–pathogen networks and global change. Trends Ecol. Evol..

[60] Seebens H, Bacher S, Blackburn TM, Capinha C, Dawson W, Dullinger S, Genovesi P, Hulme PE, van Kleunen M, Kühn I, Jeschke JM (2021). Projecting the continental accumulation of alien species through to 2050. Glob. Change Biol..

[61] Zhu G, Gutierrez Illan J, Looney C, Crowder DW (2020). Assessing the ecological niche and invasion potential of the Asian giant hornet. Proc. Natl. Acad. Sci..

[62] Gereys B, Coache A, Filippi G (2021). Présence en France métropolitaine d’un frelon allochtone: *Vespa **orientalis* Linnaeus, 1771 (Le Frelon oriental) (Hymenoptera, Vespidae, Vespinae). Faunitaxys.

[63] Graziani F, Cianferoni F (2021). The northernmost record of *Vespa **orientalis* Linnaeus, 1771 (Hymenoptera: Vespidae) in peninsular Italy. Revista gaditana de Entomología.

[64] Zachi M, Ruicănescu A (2021). *Vespa **orientalis*, a new alien species in Romania. Travaux du Muséum National d’Histoire Naturelle “Grigore Antipa”.

[65] Cini A, Santosuosso U, Papini A (2019). Uncovering the spatial pattern of invasion of the honeybee pest small hive beetle, *Aethina*
*tumida*, in Italy. Revista Brasileira de Entomologia.

[66] Monceau K, Bonnard O, Thiéry D (2014). *Vespa **velutina*: A new invasive predator of honeybees in Europe. J. Pest Sci..

[67] Mazzei M, Cilia G, Forzan M, Lavazza A, Mutinelli F, Felicioli A (2019). Detection of replicative Kashmir bee virus and Black queen cell virus in Asian hornet *Vespa **velutina* (Lepelieter 1836) in Italy. Sci. Rep..

[68] Al Naggar Y, Paxton RJ (2020). Mode of transmission determines the virulence of black queen cell virus in adult honey bees, posing a future threat to bees and apiculture. Viruses.

[69] Erenler HE, Gillman MP, Ollerton J (2020). Impact of extreme events on pollinator assemblages. Curr. Opin. Insect Sci..

[70] Nicholson CC, Egan PA (2020). Natural hazard threats to pollinators and pollination. Glob. Change Biol..

[71] Guralnick RP, Campbell LP, Belitz MW (2023). Weather anomalies more important than climate means in driving insect phenology. Commun. Biol..

[72] Martinet B, Zambra E, Przybyla K, Lecocq T, Anselmo A, Nonclercq D, Rasmont P, Michez D, Hennebert E (2021). Mating under climate change: Impact of simulated heatwaves on the reproduction of model pollinators. Funct. Ecol..

[73] Sutton TL, DeGabriel JL, Riegler M, Cook JM (2018). A temperate pollinator with high thermal tolerance is still susceptible to heat events predicted under future climate change. Ecol. Entomol..

[74] Morawetz L, Köglberger H, Griesbacher A, Derakhshifar I, Crailsheim K, Brodschneider R, Moosbeckhofer R (2019). Health status of honey bee colonies (*Apis mellifera*) and disease-related risk factors for colony losses in Austria. PLoS ONE.

[75] Brodschneider (2018). Multi-country loss rates of honey bee colonies during winter 2016/2017 from the COLOSS survey. J. Apic. Res..

[76] Kagiali E, Kokoli M, Vardakas P, Goras G, Hatjina F, Patalano S (2023). Four-year overview of winter colony losses in Greece: citizen science evidence that transitioning to organic beekeeping practices reduces colony losses. Insects.

[77] Jacques A, Laurent M, Ribière-Chabert M, Saussac M, Bougeard S, Budge GE, Hendrikx P, Chauzat MP, Epilobee Consortium (2017). A pan-European epidemiological study reveals honey bee colony survival depends on beekeeper education and disease control. PLoS ONE.

[78] Nanetti A, Bortolotti L, Cilia G (2021). Pathogens spillover from honey bees to other arthropods. Pathogens.

[79] de Souza Machado AA, Kloas W, Zarfl C, Hempel S, Rillig MC (2018). Microplastics as an emerging threat to terrestrial ecosystems. Glob. Change Biol..

[80] Yu L, Zhang J, Liu Y, Chen L, Tao S, Liu W (2021). Distribution characteristics of microplastics in agricultural soils from the largest vegetable production base in China. Sci. Total Environ..

[81] Buteler M, Alma AM, Stadler T, Gingold AC, Manattini MC, Lozada M (2022). Acute toxicity of microplastic fibers to honeybees and effects on foraging behavior. Sci. Total Environ..

[82] Wang K, Li J, Zhao L, Mu X, Wang C, Wang M, Xue X, Qi S, Wu L (2021). Gut microbiota protects honey bees (*Apis mellifera* L.) against polystyrene microplastics exposure risks. J. Hazard. Mater..

[83] Balzani P, Galeotti G, Scheggi S, Masoni A, Santini G, Baracchi D (2022). Acute and chronic ingestion of polyethylene (PE) microplastics has mild effects on honey bee health and cognition. Environ. Pollut..

[84] Al Naggar Y, Sayes CM, Collom C, Ayorinde T, Qi S, El-Seedi HR, Paxton RJ, Wang K (2023). Chronic exposure to polystyrene microplastic fragments has no effect on honey bee survival but reduces feeding rate and body weight. Toxics.

[85] Deng Y, Jiang X, Zhao H, Yang S, Gao J, Wu Y, Diao Q, Hou C (2021). Microplastic polystyrene ingestion promotes the susceptibility of honeybee to viral infection. Environ. Sci. Technol..

[86] Oliveira M, Ameixa OM, Soares AM (2019). Are ecosystem services provided by insects “bugged” by micro (nano) plastics?. TrAC Trends Anal. Chem..

[87] Anbumani S, Kakkar P (2018). Ecotoxicological effects of microplastics on biota: A review. Environ. Sci. Pollut. Res..

[88] Al Naggar Y, Brinkmann M, Sayes CM, Al-Kahtani SN, Dar SA, El-Seedi HR, Grünewald B, Giesy JP (2021). Are honey bees at risk from microplastics?. Toxics.

[89] European Commission. Commission Regulation (EU) 2022/1438 of 31 August 2022 amending Annex II to Regulation (EC) No 1107/2009 of the European Parliament and of the Council as regards specific criteria for the approval of active substances that are micro-organisms (2022).

[90] Organisation for Economic Co-operation and Development (2018). Working Document on the Risk Assessment of Secondary Metabolites of Microbial Biocontrol Agents.

[91] European Commission. Guidance on the risk assessment of metabolites produced by microorganisms used as plant protection active substances, in accordance with article 77 of Regulation (EC) No 1107/2009 (2020).

[92] European Commission. Commission Implementing Decision (EU) 2019/974 of 12 June 2019 approving the national programmes to improve the production and marketing of apiculture products submitted by the Member States under Regulation (EU) No 1308/2013 of the European Parliament and of the Council (notified under document C(2019) 4177) (2019).

[93] Martínez-López, V., Ruiz, C., & De la Rúa, P. Migratory beekeeping and its influence on the prevalence and dispersal of pathogens to managed and wild bees. *Int. J. Parasitol. Parasites Wildl.* (2022). 10.1016/j.ijppaw.2022.05.004 PMC9160285 35663725

[94] Bartlett LJ, Rozins C, Brosi BJ, Delaplane KS, de Roode JC, White A, Wilfert L, Boots M (2019). Industrial bees: The impact of apicultural intensification on local disease prevalence. J. Appl. Ecol..

[95] Ellis JS, Soland-Reckeweg G, Buswell VG, Huml JV, Brown A, Knight ME (2018). Introgression in native populations of *Apis mellifera **mellifera* L: Implications for conservation. J. Insect Conserv..

[96] Jara L, Ruiz C, Martín-Hernández R, Muñoz I, Higes M, Serrano J, De la Rúa P (2020). The effect of migratory beekeeping on the infestation rate of parasites in honey bee (*Apis mellifera*) colonies and on their genetic variability. Microorganisms.

[97] Kükrer M, Kence M, Kence A (2021). Honey bee diversity is swayed by migratory beekeeping and trade despite conservation practices: Genetic evidence for the impact of anthropogenic factors on population structure. Front. Ecol. Evol..

[98] Chen C, Parejo M, Momeni J, Langa J, Nielsen RO, Shi W, Vingborg R, Kryger P, Bouga M, Estonba A, Smartbees WP3 Diversity Contributors (2022). Population structure and diversity in European honey bees (*Apis mellifera* L.)—An empirical comparison of pool and individual whole-genome sequencing. Genes.

[99] Knapp JL, Bartlett LJ, Osborne JL (2017). Re-evaluating strategies for pollinator-dependent crops: How useful is parthenocarpy?. J. Appl. Ecol..

[100] An C, Sun C, Li N, Huang B, Jiang J, Shen Y, Wang C, Zhao X, Cui B, Wang C, Li X (2022). Nanomaterials and nanotechnology for the delivery of agrochemicals: Strategies towards sustainable agriculture. J. Nanobiotechnol..

[101] Hooven, L. A., Chakrabarti, P., Harper, B. J., Sagili, R. R., & Harper, S. L. Potential risk to pollinators from nanotechnology-based pesticides. In *Molecules*, Vol. 24, Issue 24, MDPI AG (2019). 10.3390/molecules24244458 PMC6943562 31817417

[102] Chaud M, Souto EB, Zielinska A, Severino P, Batain F, Oliveira-Junior J, Alves T (2021). Nanopesticides in agriculture: Benefits and challenge in agricultural productivity, toxicological risks to human health and environment. Toxics.

[103] Meyer WL, Gurman P, Stelinski LL, Elman NM (2015). Functional nano-dispensers (FNDs) for delivery of insecticides against phytopathogen vectors. Green Chem..

[104] Oliveira CR, Domingues CE, de Melo NF, Roat TC, Malaspina O, Jones-Costa M, Silva-Zacarin EC, Fraceto LF (2019). Nanopesticide based on botanical insecticide pyrethrum and its potential effects on honeybees. Chemosphere.

[105] Sun H (2019). Grand challenges in environmental nanotechnology. Front. Nanotechnol..

[106] Tamburini G, Bommarco R, Wanger TC, Kremen C, Van Der Heijden MG, Liebman M, Hallin S (2020). Agricultural diversification promotes multiple ecosystem services without compromising yield. Sci. Adv..

[107] Nicholson CC, Williams NM (2021). Cropland heterogeneity drives frequency and intensity of pesticide use. Environ. Res. Lett..

[108] Nichols RN, Wood TJ, Holland JM, Goulson D (2022). Role of management in the long-term provision of floral resources on farmland. Agric. Ecosyst. Environ..

[109] Péntek-Zakar E, Oleksa A, Borowik T, Kusza S (2015). Population structure of honey bees in the Carpathian Basin (Hungary) confirms introgression from surrounding subspecies. Ecol. Evol..

[110] Hatjina F, Gajda A, Dar SA (2019). Current drivers of taxonomic biodiversity loss in Asian and European bees. Phylogenetics of Bees.

[111] European Parliament. Regulation (EU) 2017/625 of the European Parliament and of the Council of 15 March 2017 on official controls and other official activities performed to ensure the application of food and feed law, rules on animal health and welfare, plant health and plant protection products, amending Regulations (EC) No 999/2001, (EC) No 396/2005, (EC) No 1069/2009, (EC) No 1107/2009, (EU) No 1151/2012, (EU) No 652/2014, (EU) 2016/429 and (EU) 2016/2031 of the European Parliament and of the Council, Council Regulations (EC) No 1/2005 and (EC) No 1099/2009 and Council Directives 98/58/EC, 1999/74/EC, 2007/43/EC, 2008/119/EC and 2008/120/EC, and repealing Regulations (EC) No 854/2004 and (EC) No 882/2004 of the European Parliament and of the Council, Council Directives 89/608/EEC, 89/662/EEC, 90/425/EEC, 91/496/EEC, 96/23/EC, 96/93/EC and 97/78/EC and Council Decision 92/438/EEC (Official Controls Regulation)European Parliament-Prospects and challenges for the EU apiculture sector-European Parliament resolution of 1 March 2018 on prospects and challenges for the EU apiculture sector, P8_TA(2018)0057, (2017/2115(INI)), (2019/C 129/05) (2017).

[112] European Commission. COMMISSION IMPLEMENTING REGULATION (EU) 2021/632 of 13 April 2021 laying down rules for the application of Regulation (EU) 2017/625 of the European Parliament and of the Council as regards the lists of animals, products of animal origin, germinal products, animal by-products and derived products, composite products, and hay and straw subject to official controls at border control posts, and repealing Commission Implementing Regulation (EU) 2019/2007 and Commission Decision 2007/275/EC (2021).

[113] European Commission. Commission Implementing Regulation (EU) 2021/403 of 24 March 2021 laying down rules for the application of Regulations (EU) 2016/429 and (EU) 2017/625 of the European Parliament and of the Council as regards model animal health certificates and model animal health/official certificates, for the entry into the Union and movements between Member States of consignments of certain categories of terrestrial animals and germinal products thereof, official certification regarding such certificates and repealing Decision 2010/470/EU (2021).

[114] European Commission. COMMISSION DELEGATED REGULATION (EU) 2020/692 of 30 January 2020 supplementing Regulation (EU) 2016/429 of the European Parliament and of the Council as regards rules for entry into the Union, and the movement and handling after entry of consignments of certain animals, germinal products, and products of animal origin (2020).

[115] European Commission. Commission acts for global food security and for supporting EU farmers and consumers. Press Release, Brussels, 23.3.2022 (2022).

[116] Harris, C., & Ratnieks, F. L. Clover in agriculture: combined benefits for bees, environment, and farmer. *J. Insect Conserv.* 1–19 (2022).

[117] European Commission. EU Pesticides Database. (2022) https://food.ec.europa.eu/plants/pesticides/eu-pesticides-database_en.

[118] European Parliament. Regulation (EC) No 1185/2009 of the European Parliament and of the Council of 25 November 2009 concerning statistics on pesticides (2009).

[119] Laaninen, T. New genomic techniques: European Commission study and first reactions (Briefing-European Parliamentary Research Service, 2021).

[120] Jinek M, Chylinski K, Fonfara I, Hauer M, Doudna JA, Charpentier E (2012). A programmable dual-RNA-guided DNA endonuclease in adaptive bacterial immunity. Science.

[121] Eisenstein M (2022). Base edit your way to better crops. Nature.

[122] Kumar K, Gambhir G, Dass A, Tripathi AK, Singh A, Jha AK, Yadava P, Choudhary M, Rakshit S (2020). Genetically modified crops: Current status and future prospects. Planta.

[123] Malone, L. A., & Burgess, E. P. J. Impact of genetically modified crops on pollinators. In *Environmental Impact of Genetically Modified Crops, *199–222 (CAB International, 2009).

[124] Brookes G, Barfoot P (2018). Environmental impacts of genetically modified (GM) crop use 1996–2016: Impacts on pesticide use and carbon emissions. GM Crops Food.

[125] Roy DB, Bohan DA, Haughton AJ, Hill MO, Osborne JL, Clark SJ, Perry JN, Rothery P, Scott RJ, Brooks DR (2003). Invertebrates and vegetation of field margins adjacent to crops subject to contrasting herbicide regimes in the farm scale evaluations of genetically modified herbicide–tolerant crops. Philos. Trans. R. Soc. Lond. Ser. B Biol. Sci..

[126] Balfour N, Ratnieks F (2022). The disproportionate value of ‘weeds’ to pollinators and biodiversity. J. Appl. Ecol..

[127] European Commission. Directorate-General for Communication, *Circular economy action plan: for a cleaner and more competitive Europe*, Publications Office of the European Union. https://data.europa.eu10.2779/05068 (2020).

