# Supplementary material for: Emerging threats and opportunities to managed bee species in European agricultural systems: a horizon scan
Source: Sci Rep. 2023 Oct 23;13:18099. doi: 10.1038/s41598-023-45279-w (PMC10593766; doi:10.1038/s41598-023-45279-w)
Supplement: Supplementary file 1 — Supplementary Table 1. [file 41598_2023_45279_MOESM1_ESM.docx]

**Emerging threats and opportunities to managed bee species in European agricultural systems - A Horizon Scan**

Bryony K. Willcox^1*^, Simon G. Potts^1^, Mark J.F. Brown^2^, Anne Alix^3^, Yahya Al Naggar^4,5,6^, Marie-Pierre Chauzat^7^, Cecilia Costa^8^, Antoine Gekière ^9^, Chris Hartfield^10^, Fani Hatjina^11^, Jessica L. Knapp^12,13^, Vicente Martínez-López^14,15^, Christian Maus^16^, Teodor Metodiev^17^, Francesco Nazzi^18^, Julia Osterman^19,20,21^, Risto Raimets^22^, Verena Strobl^23^, Annette Van Oystaeyen^24^, Dimitry Wintermantel^19^, Nikol Yovcheva^17^ and Deepa Senapathi^1^

**SI Table 1:** The full list of 63 issues identified as a part of our 2022 Horizon Scan process. Issues in bold are the final 21 prioritized issues.

|  |  | **Median Rank** |
| --- | --- | --- |
| **Issue #** | **Topic** | **1^st^ round scoring** |
| 1 | Densely populated areas - concentrating large number of honey bee colonies in certain areas will be a good breeding ground for the spread of diseases and parasites | 25.5 |
| 2 | The effect of protective covers on managed pollinators | 27.5 |
| **3** | **Increasing threat of emerging pathogens and predators** | **10** |
| **4** | **Increase of varroa-resistant stocks of *Apis mellifera*** | **17.5** |
| 5 | Impact of broad-spectrum insecticides (such as pyrethroids, organophosphates and carbamates) being used more widely in the wake of the ban on neonicotinoids | 35.5 |
| **6** | **Prime editing and genetically modified crops in Europe** | **23** |
| 7 | Use the eco-exposome concept to protect pollinators | 38 |
| **8** | **Exposure to micro or nano plastics either alone or in combination with other stressors and transgenerational impacts on bees and bee health** | **20.5** |
| 9 | Develop monitoring and analytical tools to identify and monitor microplastic pollution in the environment | 33 |
| **10** | **Cutting pollinators out of food production** | **12.5** |
| 11 | The replacement of bees with drones, robotic bees, AI | 38 |
| **12** | **Direct or indirect effects of biopesticides on bees** | **20.5** |
| **13** | **Increase of migratory beekeeping** | **11.5** |
| **14** | **Increase of inexperienced beekeepers** | **22.5** |
| **15** | **Extreme weather events** | **10.5** |
| **16** | **Impact of Ukraine Invasion on the EU Common Agricultural Policy (Rapid policy changes or delay of the green-deal due to Russian attack on Ukraine), food prices and agroecological transitions** | **20.5** |
| 17 | Increased environmental pollution/ pollinators health and human health (heavy metals & nanomaterials) | 24 |
| 18 | Wild bees as managed pollinators | 40 |
| 19 | Emerging technologies: telecommunication networks 5G (6, 7G) and the impact of electromagnetic fields / high-frequency radiation on bee health | 40 |
| 20 | Impact of covid and resulting quarantine and cross border restrictions on commercial beekeeping and health of managed pollinators | 53 |
| **21** | **Strengthening trade and biosecurity measures in the EU to better protect local managed bee populations, managed bee breeding and trade.** | **16.5** |
| 22 | Companion cropping and oilseed rape | 34 |
| 23 | What are the impacts of increased technology use in monitoring bee colonies remotely? (e.g., energy consumption concerns, loss of field expertise) | 37 |
| **24** | **Greater availability of technology and automation to remotely monitor bee colony health.** | **21** |
| 25 | Exposure assessment for precision application methods | 30 |
| 26 | New, increasingly user-friendly bee models allow us to generalise ecological patterns and ask questions beyond our empirical abilities | 26 |
| 27 | How do poor regulations surrounding bee hotels and their design, impact on bee health? | 49 |
| 28 | Landscape simplification and the profitability of unifloral honeys, influencing the beekeeper‘s managing strategies | 24 |
| **29** | **Artificial intelligence for disease, weed and pest control to reduce pesticide use in agroecosystems** | **23** |
| 30 | Africanized bees and the negative behavioural traits associated with them | 48 |
| 31 | Land abandonment of extensive farmlands and agroforestry systems leading to landscape and vegetation homogenisation which are known to have negative consequences for biodiversity conservation | 39.5 |
| 32 | Strengthen the environmental responsibility: protect the pollinators by law using the crime of ecocide | 39 |
| 33 | Producing bumble bee colonies on demand. Will Biobest, Koppert, and other companies be able to respond to changing crop phenology from climate change. Potentially leading to the over-production of bumblebee colonies and, thus, ethical issues | 40 |
| 34 | Declining air quality (pollution) and the interaction with climate change | 24.5 |
| **35** | **Thermic vehicles and the hazardous pollutants they release will decrease in the coming years, does switching to electric vehicles represent an opportunity for managed bees?** | **22.5** |
| 36 | How do we address the lack of effective and affordable anti-parasitic and veterinary treatments for managed bees? | 29 |
| 37 | Pesticides: Risk of exposure from greenhouses | 31 |
| **38** | **Co-formulants in agrochemical formulations and managed bee health.** | **9.5** |
| **39** | **Optimising diets of managed bees to develop better artificial diets and inform agri-environment schemes** | **16** |
| **40** | **Agricultural policy to encourage biodiversity-promoting floral resources on arable land** | **20.5** |
| **41** | **Accessibility of European pesticide exposure datasets** | **27** |
| 42 | Include testing pesticide side-effects on pollinators in target crops as a requirement in the EU guidelines | 27.5 |
| 43 | Non-destructive DNA sampling for conservation using non-lethal sampling methods like tarsal clips, swabbing or airborne eDNA | 36.5 |
| **44** | **Changing farm practice and timing of the demand for managed bees** | **21** |
| 45 | Development of undetectable methods of adulteration of bee products and production of synthetic products (vegan products) | 38 |
| 46 | Farm-to-Fork strategy and food industry: food industry and retailers as a driver towards pesticide reduction | 25 |
| 47 | Disagreements between beekeeping and wild pollinator conservation groups have potential to spill over into public sphere – through media – might lead to erosion of public trust | 35 |
| 48 | Apis mellifera: spread of unmanaged colonies as an opportunity | 29 |
| 49 | Opportunities to utilise manage pollinators (bumbles and solitaries) in allotments to improve food security and self-sufficiency of urban agriculture | 39.5 |
| 50 | Using plant secondary chemicals to optimise managed bumble bee health | 24 |
| **51** | **Nanotechnology-based pesticides (NBPs)** | **16** |
| 52 | Issue of bee bycatch in pheromone traps | 48 |
| 53 | New spaces for honey bees – solar parks | 24 |
| 54 | Decrease of bee fitness from impacts of multiple stressors (chemical/biological/nutritional) on bee microbiome | 31 |
| 55 | Competition for resources and impacts on health of managed bees | 39 |
| 56 | Silage Crops: A future increase of the cultivation of silage crops could further reduce the availability of forage for managed pollinators in the agricultural landscape | 41 |
| 57 | Tendency to destroy old building that may be suitable habitats for Osmia and other managed cavity-nesting bees | 53 |
| **58** | **Development of field instruments for evaluation of genetic markers to be used in breeding for resilience.** | **21** |
| 59 | Educating younger generation about preserving managed pollinators | 27.5 |
| 60 | Could managed solitary bees be invasive? | 38 |
| 61 | Carbon farming | 36 |
| 62 | Increasing prevalence of artificial grass in urban areas | 41 |
| 63 | Opportunities for Certification for pollinator friendly products and good practices | 31.5 |
